# A data-driven framework to assess population dynamics during novel coronavirus outbreaks: A case study on Xiamen Island, China

**DOI:** 10.1371/journal.pone.0293803

**Published:** 2023-11-10

**Authors:** Peng Wang, Jinliang Huang

**Affiliations:** Fujian Key Laboratory of Coastal Pollution Prevention and Control, Xiamen University, Xiamen, China; The University of Hong Kong, HONG KONG

## Abstract

The outbreak of the Coronavirus Disease 2019 (COVID-19) has profoundly influenced daily life, necessitating the understanding of the relationship between the epidemic’s progression and population dynamics. In this study, we present a data-driven framework that integrates GIS-based data mining technology and a Susceptible, Exposed, Infected and Recovered (SEIR) model. This approach helps delineate population dynamics at the grid and community scales and analyze the impacts of government policies, urban functional areas, and intercity flows on population dynamics during the pandemic. Xiamen Island was selected as a case study to validate the effectiveness of the data-driven framework. The results of the high/low cluster analysis provide 99% certainty (P < 0.01) that the population distribution between January 23 and March 16, 2020, was not random, a phenomenon referred to as high-value clustering. The SEIR model predicts that a ten-day delay in implementing a lockdown policy during an epidemic can lead to a significant increase in the number of individuals infected by the virus. Throughout the epidemic prevention and control period (January 23 to February 21, 2020), residential and transportation areas housed more residents. After the resumption of regular activities, the population was mainly concentrated in residential, industrial, and transportation, as well as road facility areas. Notably, the migration patterns into and out of Xiamen were primarily centered on neighboring cities both before and after the outbreak. However, migration indices from cities outside the affected province drastically decreased and approached zero following the COVID-19 outbreak. Our findings offer new insights into the interplay between the epidemic’s development and population dynamics, which enhances the prevention and control of the coronavirus epidemic.

## 1 Introduction

Infectious diseases pose a substantial burden globally [1, 2]. An estimated 50 million individuals succumb to various infectious diseases each year [3]. The Severe Acute Respiratory Syndromes (SARS) outbreak, which started in China in early 2003, extended to more than 30 countries within three months, infecting roughly 8,422 individuals worldwide [4]. Similarly, in 2009, the H1N1 virus outbreak, which began in Mexico, spread globally and resulted in approximately 4,100 deaths within six months [5]. In December 2019, numerous cases of pneumonia of unknown cause were detected in Wuhan, Hubei Province, China. This condition was later named novel coronavirus disease (COVID-19) and is highly contagious, prompting significant international concern [6–8]. As of December 23, 2022, China reported a cumulative total of 9,558,276 confirmed cases [9]. Globally, by January 26, 2023, there have been 665,078,673 diagnosed cases and 6,725,248 reported deaths [10]. The COVID-19 pandemic has brought about profound global-scale disruptions to daily life, social dynamics, and economic development [11–14].

Effective control of COVID-19 spread is of paramount importance. Measures such as vaccination, herd immunization, and lockdowns have been utilized to curb the pandemic [15–17]; however, the disparate availability and affordability of vaccines across various populations have significantly influenced the outcomes. Allowing populations to develop natural herd immunity has incited controversy due to the potential to exacerbate already strained healthcare systems, which could potentially increase fatalities [11].

Most countries have instituted lockdown measures to prevent and control the virus spread, however, the detrimental effects of prolonged lockdowns on socioeconomic factors and everyday life cannot be ignored [11, 18–21]. Several studies have drawn a connection between population movement and the spread of COVID-19 [22–24]. Therefore, understanding the shifts in human mobility during a pandemic and how it reacts to various directives is of vital importance. However, acquiring this understanding is challenging due to limited data and the presence of complex confounding effects. Earlier research has suggested the feasibility of collecting and closely monitoring human mobility data through platforms such as mobile phone signaling, Twitter, Weibo, WeChat, and other social media [25–32]. Data from social media have been extensively used to probe the relationship between population dynamics and the virus propagation rate [33–35].

Understanding the spatiotemporal distribution of a population is vital for the prevention and control of COVID-19 [36–39]. COVID-19 cases exhibit multiscale characteristics, and the spread of infection shows a spatial association with population density [40, 41]. Throughout the COVID-19 outbreak, research on population dynamics employed data from multiple sources [35–40]. With its high temporal resolution, the Baidu population heatmap is particularly suited for studying urban population dynamics [42]. In spatial analyses of epidemics, scale is a pivotal factor. The sporadic nature of COVID-19 infection and its temporal shifts have been examined on the country and continental scales [40]. Moreover, research on the majority of the population and COVID-19 has primarily been conducted at the global, national, city, and county levels [7, 22, 43, 44]. Nevertheless, controlling the spread of the virus at smaller spatial scales, such as communities, streets, and buildings, can be instrumental in minimizing the adverse impacts of virus transmission.

Investigating the influencing factors of population dynamics during an epidemic can facilitate the formulation of preventative and control policies. However, numerous intricate factors affect population dynamics. For instance, previous research has indicated that elements such as globalization, characteristics of human settlements, demographic attributes, and high population mobility contribute to disease spread [45]. Additionally, epidemic prevention policies, urban functional areas, and intercity flows impact population dynamics [7, 42, 46–50]. Policies such as home quarantine and remote work have substantially mitigated the number of infected individuals and influenced population dynamics [51, 52]. Comprehensive research on the implementation of epidemic prevention policies for population prediction and dynamics of COVID-19 infection has yielded remarkable results [53–55]. Models such as the Susceptible, Exposed, Infected, and Recovered (SEIR) and modified SEIR are widely utilized to predict the number of infectious disease cases [56–58]. Furthermore, deep learning techniques are frequently applied in studies of population dynamics [51, 59–66]. Different urban functional areas exhibit varying impacts on population densities and dynamics [42]. For instance, commercial zones tend to experience high population mobility and concentration. Urban functional zoning based on points of interest (POI) is an effective strategy that outperforms traditional methods [67]. Cities display distinct characteristics of population flow owing to economic, cultural, and resource factors. These differences reflect diverse population dynamics at various stages of an epidemic [68, 69].

Understanding population dynamics during a pandemic and the response to various directives is critical for developing effective policies to control virus spread and mitigate social, economic, and human impacts. In this study, we present a data-driven framework to examine population dynamics during the COVID-19 outbreak on Xiamen Island, southeast coast of China. The objectives of this study are as follows: (1) to visualize and analyze population dynamics during the COVID-19 pandemic using the Baidu population heatmap data and (2) to identify the influential factors on population dynamics during the pandemic. The insights gained from this study are a valuable reference for policymakers confronting novel coronavirus outbreaks.

## 2 Data and methodology

### 2.1 Study area

Xiamen Island, located on the southeast coast of Fujian Province, has a resident population of approximately 2.08 million, spans a land area of about 158 km^2^, and consists of two main districts, namely Huli and Siming (**Fig A1 in S1 Appendix**). The Huli District, located in the north of Xiamen Island, encompasses various subdistricts, such as Jiangtou, Heshan, Huli, Jinshan, and Dianqian. Known for its thriving commercial spaces, residential neighborhoods, and industrial zones, Huli significantly contributes to the island’s economy. Conversely, the Siming District is situated in the southern part of Xiamen Island and comprises multiple subdistricts, including Kaiyuan, Jialian, Zhonghua, Yuandang, Xiagang, Wucun, Lujiang, Lianqian, and Binhai. The Siming District is renowned for its bustling commercial hubs, picturesque coastal scenery, and historical landmarks. In the Huli District, the Jinshan subdistrict lies in the eastern part, while in the Siming District, the Binhai and Lianqian subdistricts occupy a substantial part of the east and south regions. Lujiang and Xiagang subdistricts are situated in the western region of the Siming District. Wucun, Jiangtou, and Jialian subdistricts are positioned centrally on Xiamen Island. The Island exhibits high levels of population aggregation and mobility [70]. Fig A2 in S1 Appendix presents a heatmap illustrating the population distribution on Xiamen Island and its surrounding areas.

### 2.2 Method

**Fig 1:**
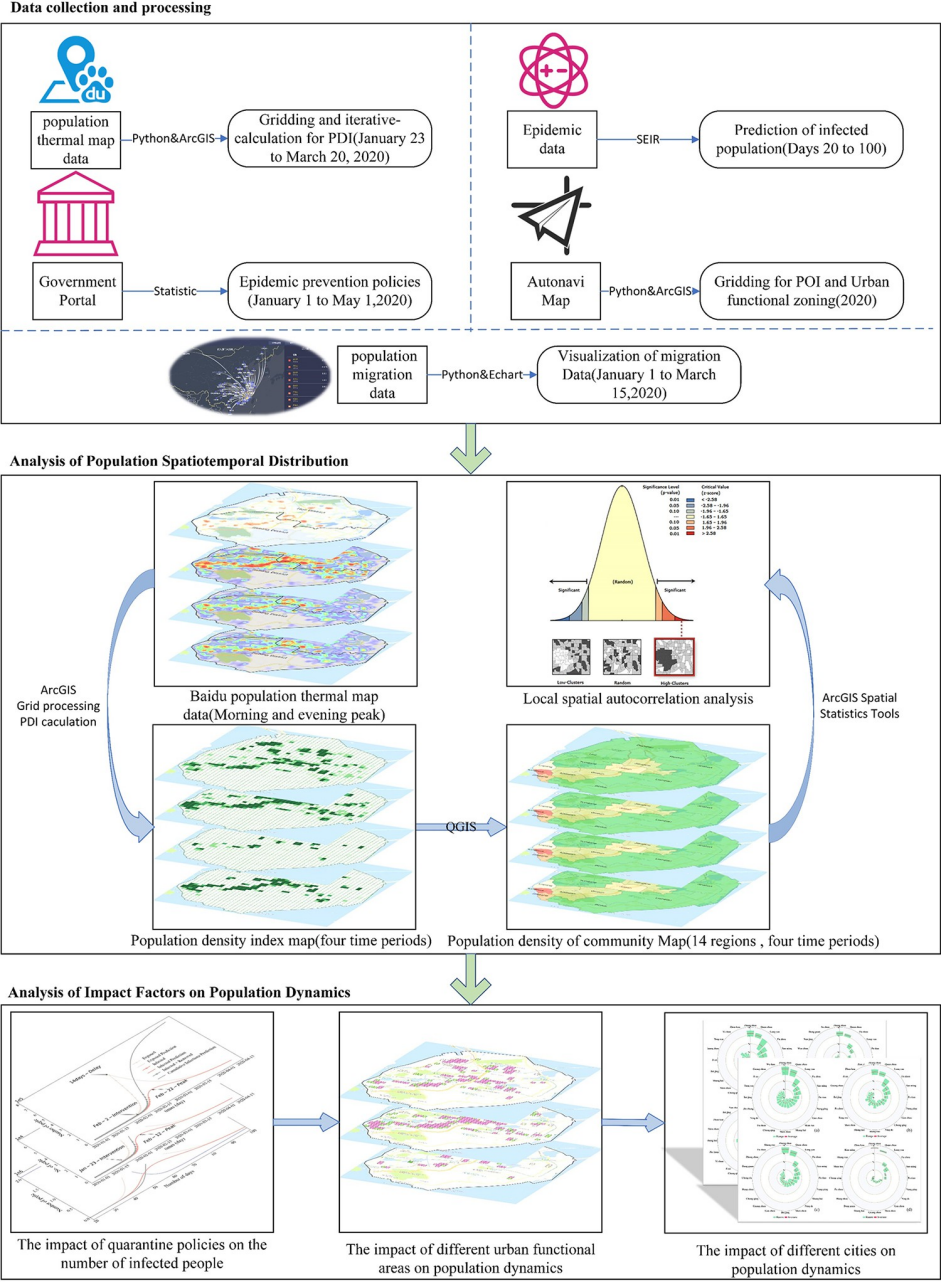
Diagram illustrating the proposed approach’s framework.

The methodology employed in this study is presented in **Fig 1**, which comprises the following stages: (1) data collection and processing, (2) analysis of population spatiotemporal distribution, and (3) analysis of impact factors on population dynamics.

The initial stage involves data collection and processing, which gathers pertinent data to support the investigation into population dynamics within the study area. The subsequent stage scrutinizes the spatiotemporal distribution of populations, to uncover their distribution patterns during the COVID-19 epidemic. The final stage addresses the factors influencing population dynamics during the COVID-19 epidemic, targeting the exploration of the relationship between population dynamics and the epidemic’s progression.

#### 2.2.1 Analysis of population spatiotemporal distribution

The Baidu population heat maps, PDI, and community-level population density were used to depict population distribution across different epidemic stages. Local spatial autocorrelation analysis was conducted to evaluate the clustering characteristics of population distribution in the study area. Hourly data from the Baidu population thermal maps covering the entirety of Xiamen Island from January 23 to March 20, 2020 were collected. The processing of the Baidu heat map included Geo-registration, time aggregation, grid creation, and calculations of the Population Density Index (PDI) [42]. We used the WGS 84 coordinate system to align the population heat map with other spatial data, ensuring that all heat maps shared the same extent and size. PDI was employed to normalize the data, represented by the formula:

$$PDI=Q_{\mathrm{th}}/\sum Q_{\mathrm{th}}$$ (1)

where PDI is the normalized population density index, Q_th_ is the sum of the heat map values for a specific region H at a particular time t, and ∑Q_th_ is the sum of the heat map values of all regions at a certain time t.

To maximize the utility of the datasets and facilitate analysis, the data were divided into two time periods: morning peak (07:00–09:59) and evening peak (17:00–19:59). The average PDI for each period was calculated. For computational simplicity while retaining the majority of the heat map information, we constructed 1,568 spatially connected grids spanning the entire island. These grids were designed to mirror population dynamics, each with a spatial size of 300 × 300 m.

We employed the PDI index to analyze the spatial clustering of population density on Xiamen Island. The local clustering characteristics of each grid were assessed using local spatial autocorrelation analysis in ArcGIS 10.2. The Getis-ORD Gi* method was used to evaluate the local clustering characteristics of each time slot. As previously described [71, 72], the spatial and temporal distribution of the population was determined.

#### 2.2.2 Analysis of impact factors on population dynamics

We examined the impact of different epidemic prevention policies, urban functional areas, and cities on population dynamics. The SEIR model was utilized to forecast the number of infected individuals, which allowed us to assess the impact of policies on population dynamics and the spread of the epidemic [73]. We used the POI data to establish urban functional zoning and investigated how different urban functional zones influenced population dynamics during the epidemic. Intercity migration indices were computed and mapped to assess the influence of various cities on population dynamics amid the pandemic.

The SEIR model categorizes a population into susceptible (S), exposed (E), infected (I), and recovered (R) individuals, assuming that all individuals in the population are susceptible to infection. Upon recovery, individuals produce antibodies, therefore, the recovered population, denoted as R, will not be reinfected. By hypothesizing a differential change in the formula of the SEIR model, we derived the following equations:

$$S(t+1)=S(t)-\frac{\beta S(t)I(t)}{N}$$ (2)

$$E(t+1)=E(t)+\frac{\beta S(t)I(t)}{N}-\delta E(t)$$ (3)

I(t+1)=I(t)+δE(t)−γI(t) (4)

R(t+1)=γI(t)+R(t) (5)

where t is time in days, S(t) is the number of susceptible individuals in a region, βis the rate of transmission rate from susceptible to infected individuals, E(t) is the number of exposed individuals in a region, δ is the incubation rate, I(t) is the number of infected individuals in a region,γ is the recovery or mortality probability, and R(t) is the number of recovered or deceased individuals in a region.

The model’s assumptions are as follows: (1) the recovered population R produces antibodies and cannot be reinfected; (2) the natural mortality and population growth rates were not considered during the period under study; (3) the influence of asymptomatic infected individuals was not considered. The SEIR infection mechanism is depicted in **Fig A3 in S1 Appendix**.

Through calculations and referencing relevant literature, we obtained the necessary parameters for the model as follows: The total population is 2,110,289 [70]; the infection rate coefficient is 0.2443; the disease incubation period is 14 days; the initial number of infected individuals is 1; both the initial number of latent individuals and the initial number of recovered individuals are 0. The initial number of susceptible individuals is equal to the total population minus the initial number of infected, latent, and recovered individuals. The infection and recovery rates were validated using relevant statistical data from domestic provinces. Additional statistical data on SARS-CoV-2 are presented in S3.

### 2.3 Data sources

The Baidu software was used to procure population migration data for Xiamen City as well as hourly population thermal map data covering the entirety of Xiamen Island. POI data were gathered using a map from Autonavi, a subsidiary of Alibaba Group, China. Data regarding Xiamen City’s historical epidemics were sourced from the DXY Epidemic Information website [74]. Information on epidemic policies for the Fujian Province was accessed from the Fujian Health Commission’s website [75].

## 3 Results

### 3.1 Spatiotemporal distribution of population

We employed spatial mapping techniques to visualize the spatiotemporal distribution of the population using Baidu’s population heatmap data at various time intervals(**Figs A4** and **A5 in S1 Appendix**). The visual evidence in these figures shows that the population of Xiamen Island is predominantly clustered in the central, southwest, east, and north regions. These areas likely have higher population densities relative to other parts of the island, indicating that the spatial mapping method utilized in this study could be applied to other densely populated cities or countries. On Xiamen Island, the population flow and density are higher during the evening peak compared with the morning peak.

**Fig 2:**
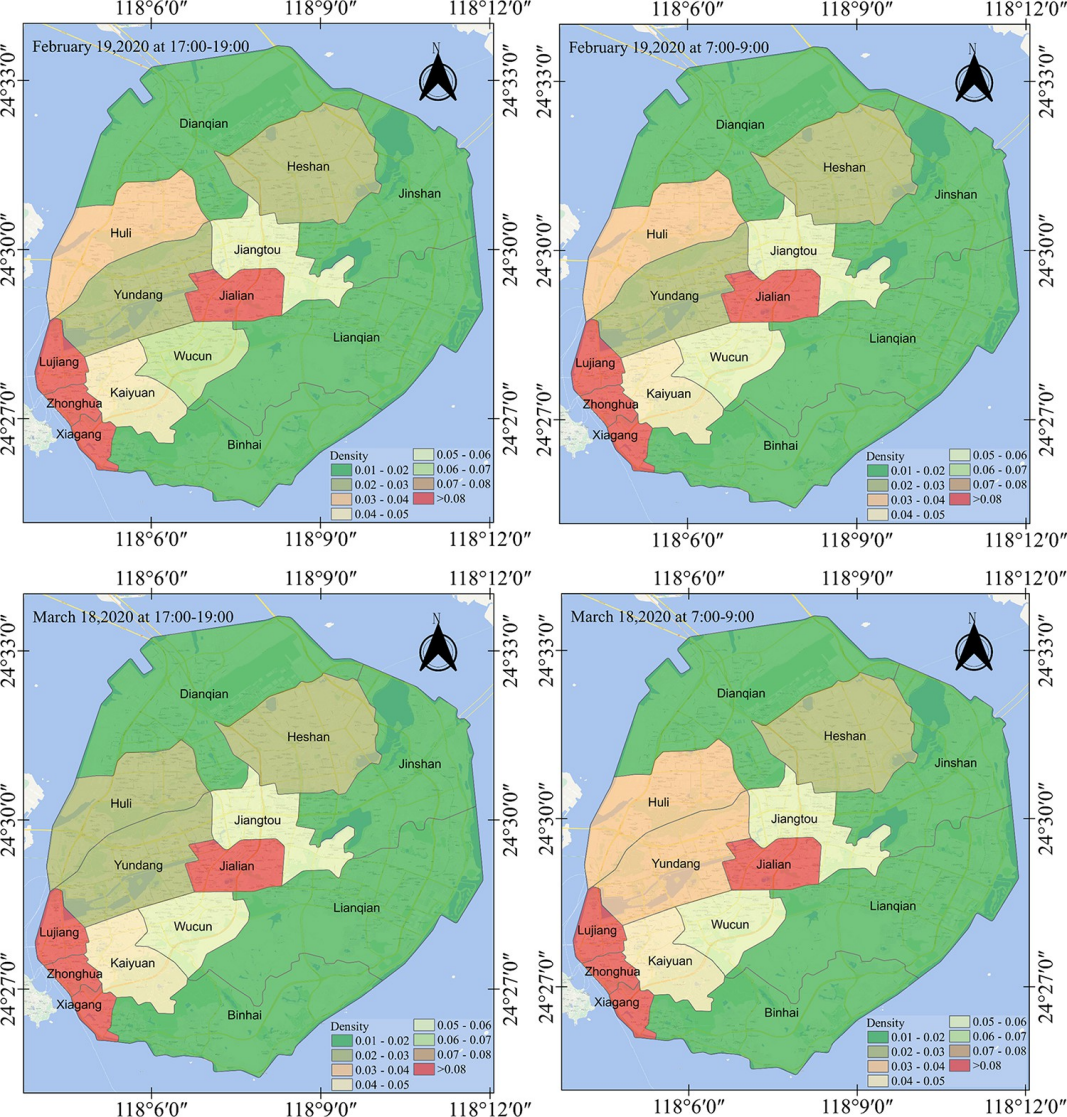
Variations in population density across different zones during distinct periods.

**Fig 2** illustrates the population distribution of community units in Xiamen Island during the novel coronavirus prevention and control period. By examining these dynamics, authorities can identify areas with elevated population densities and potentially higher transmission risks. As depicted in Fig 2, most epidemic cases in Xiamen Island were reported in the Siming and Huli districts, likely due to their significant population density. Table 1 lists the relative population densities for 14 communities on Xiamen Island. The population is primarily concentrated in the communities located in the northern part of the Siming District and the southern part of the Huli District, typically characterized as residential zones or popular tourist destinations with substantial population and movements.

**Table 1:** Comparative population density across various regions during distinct periods.

Subdistrict Name	Morning peak (Feb–19)	Morning peak (Mar–18)	Evening peak (Feb–19)	Evening peak (Mar–18)
Xiagang	0.2704	0.2753	0.2808	0.2887
Zhonghua	0.2054	0.1914	0.1932	0.1831
Binhai	0.1370	0.1464	0.1402	0.1484
Kaiyuan	0.0871	0.0881	0.0883	0.0891
Wucun	0.0597	0.0567	0.0610	0.0568
Lujiang	0.0523	0.0561	0.0546	0.0555
Jialian	0.0495	0.0454	0.0452	0.0423
Lianqian	0.0308	0.0306	0.0306	0.0296
Yundang	0.0290	0.0301	0.0289	0.0293
Jiangtou	0.0262	0.0283	0.0272	0.0282
Huli	0.0164	0.0171	0.0160	0.0165
Heshan	0.0135	0.0123	0.0120	0.0118
Jinshan	0.0120	0.0120	0.0116	0.0110
Dianqian	0.0108	0.0100	0.0103	0.0096

We employed the Population Density Index (PDI) to conduct a high-low clustering analysis, exploring the characteristics of population distribution within the study area. As illustrated in **Fig A6 in S1 Appendix** [76] and Table B1 in S2 Appendix, the population distribution is not random (P < 0.01). A calculated Z-score > 2.58, accompanied by the observation value (G) exceeding the expected value (G expected), denotes a high-value clustering of the population. The high/low cluster analysis unequivocally reveals that Xiamen Island showcases a high-density population distribution. This highlights the relevance and applicability of this method to other cities and countries with comparable urban development and population dynamics.

### 3.2 Impact factor of population dynamics

**Fig 3:**
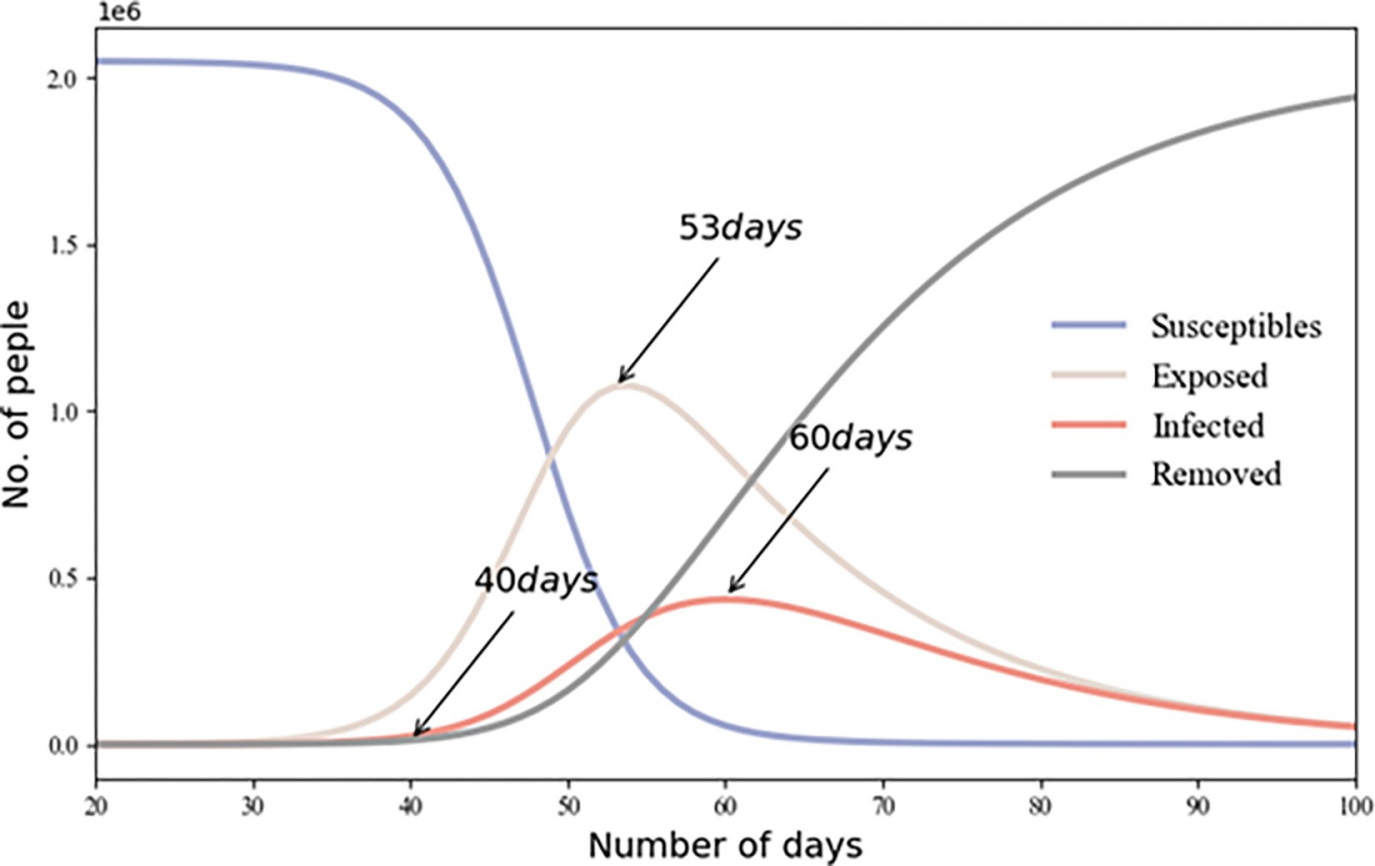
Prediction of COVID-19 progression in Xiamen using the SEIR model.

**Fig 4:**
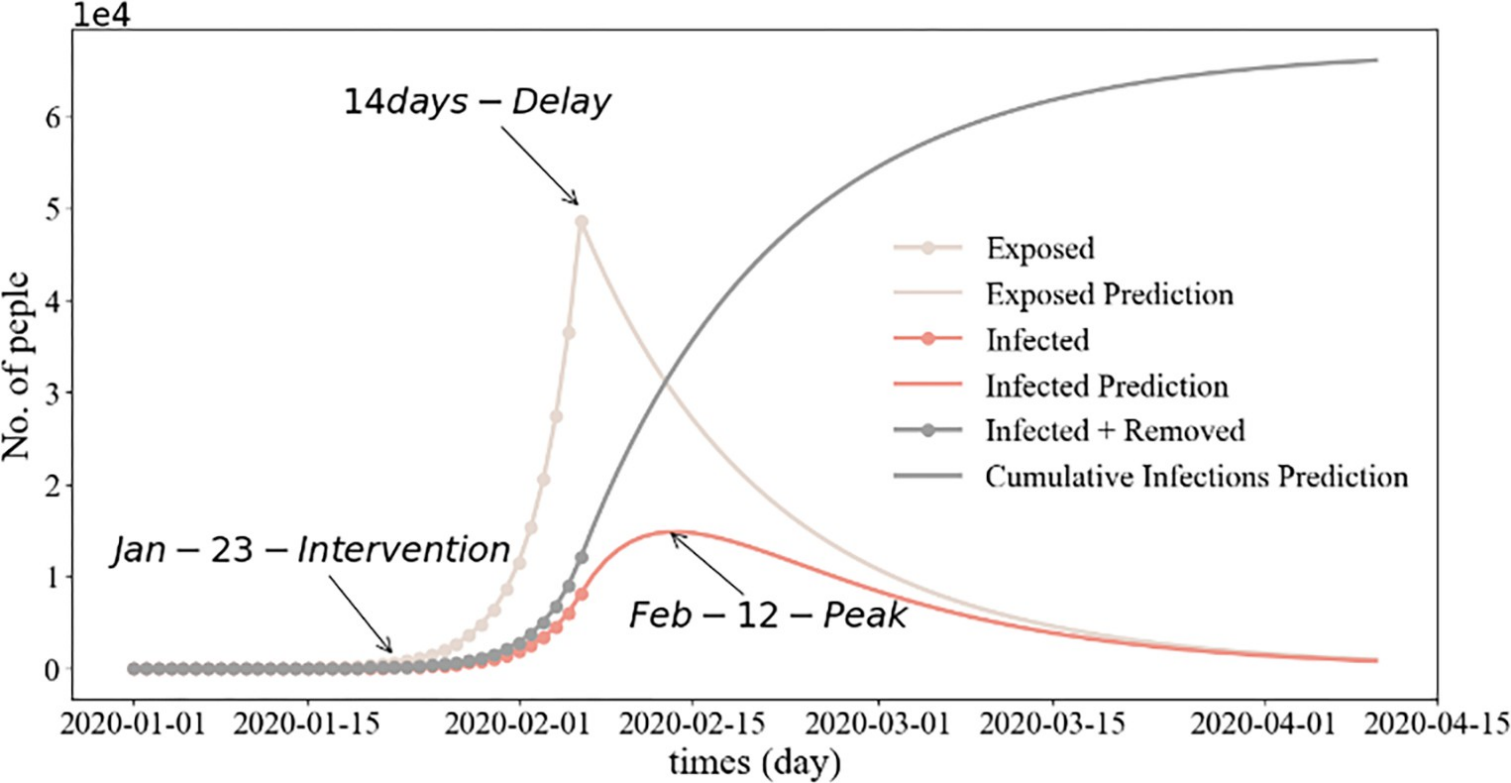
Prediction of the SEIR model for Xiamen City before and after lockdown implementation.

**Fig 5:**
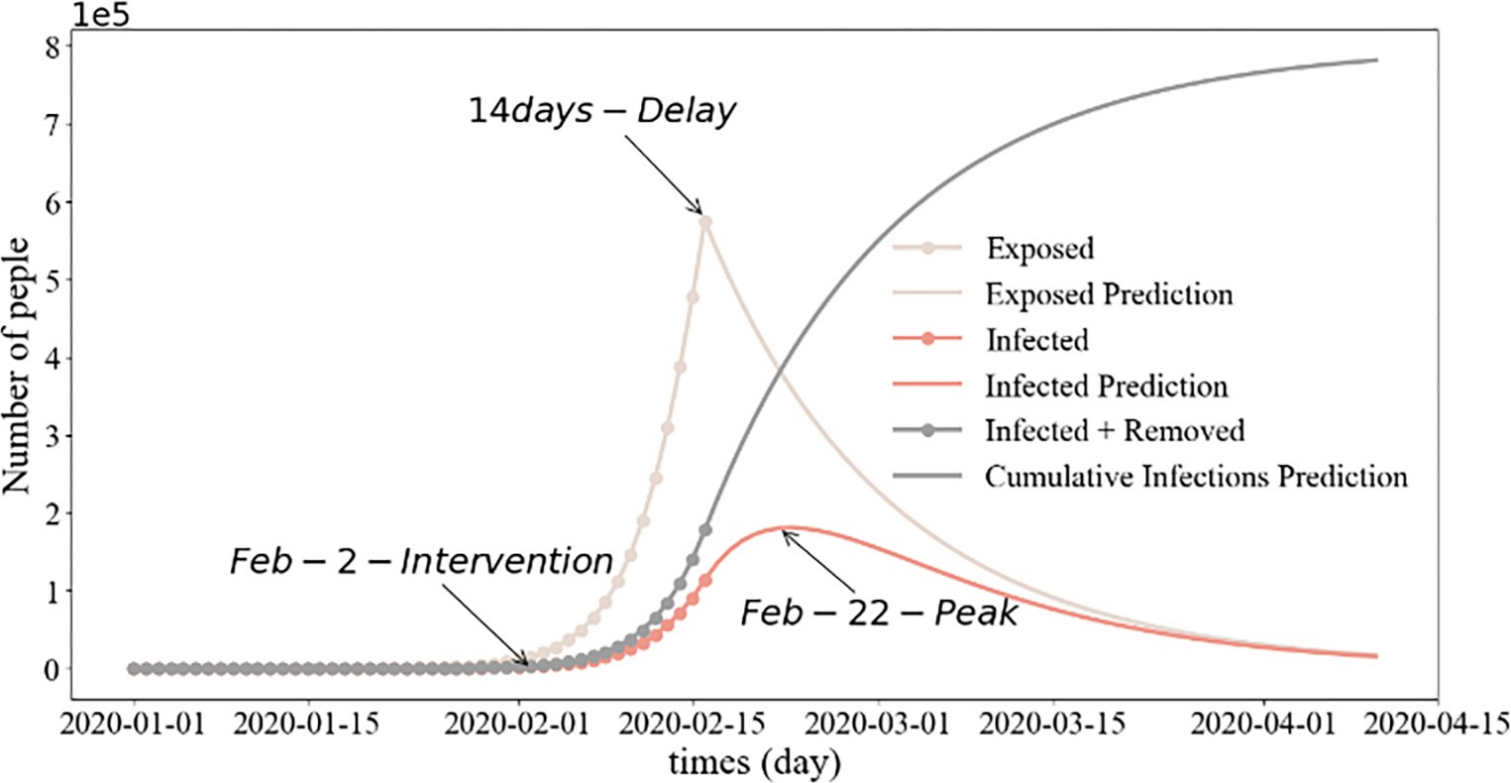
Projection of the SEIR model for Xiamen, comparing scenarios before and after the lockdown implemented on February 2.

Our investigation focused on the factors influencing population dynamics during the pandemic, with a specific focus on three key aspects, namely epidemic prevention policies, urban functional areas, and inter-city population migration indices during the epidemic. The Chinese government implemented the lockdown policy during the initial stages of the outbreak as a preventative measure against the virus spread by regulating population gatherings and movements. We used the SEIR model to simulate the epidemic progression in Xiamen. As depicted in **Fig 3**, the number of infected individuals peaked at approximately 60 days after the outbreak. According to the simulation conducted using the SEIR model, **Fig 3** shows that the number of infected individuals on Xiamen Island peaked approximately 60 days after the initial outbreak. Xiamen government enforced lockdown measures on January 23, 2020, and gradually resumed work starting on February 9 to control the virus spread. The existing SEIR model was enhanced and simulated in two phases, before and after the lockdown, on January 23 (**Fig 4**). We also simulated a scenario where the city lockdown was delayed by ten days, to February 2, 2020 (**Fig 5**). As indicated in **Figs 4** and **5**, a ten-day delay in implementing the lockdown could intensify the spread of COVID-19. The earlier the home isolation policy is enacted, the lower the number of new reported infections.

**Fig 6:**
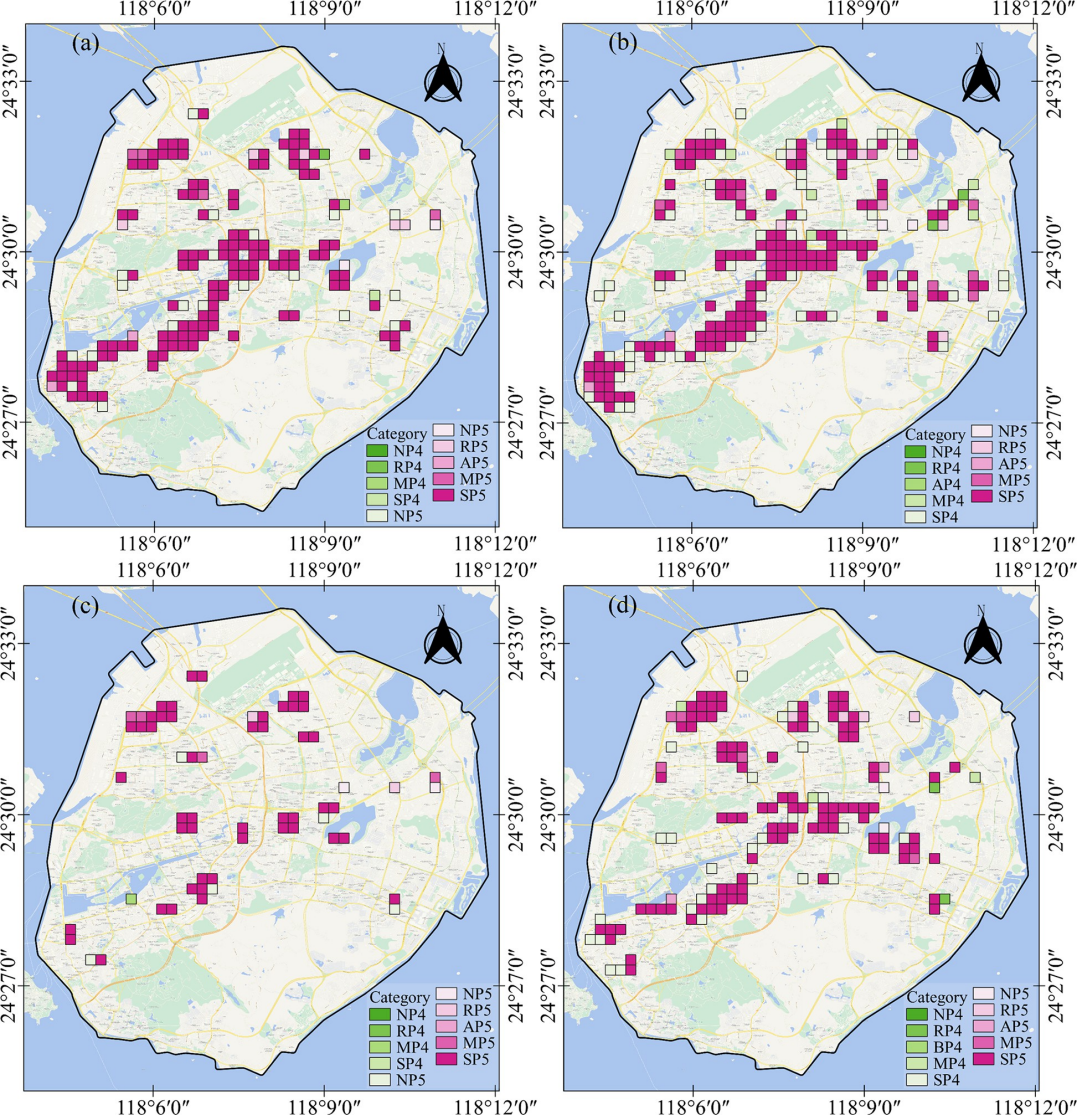
Illustration of different periods: (a) 17:00–19:59 on February 17, 2020, (b) 17:00–19:59 on March 16, 2020, (c) 7:00–8:59 on February 18, 2020, and (d) 7:00–8:59 on March 17, 2020. Key for annotations: R—residential, A -public management and public service facilities, B—commercial service facilities, M—industrial, S—transportation and road facilities, G—green space and square land, N—the lattice of POIs not to be included, P4—grid values in the range of 60–80, P5—grid values in the range of >80, AP4—grid values in the range of 60–80 of public management and public service facilities.

Urban functional areas demonstrate distinct population dynamics. For instance, shopping malls generally witness high population concentration. As depicted in **Fig A7 in S1 Appendix**, urban land use and functional area types for 1,099 grid cells on Xiamen Island are categorized into single–functional, mixed-functional, and non-construction areas. Residential areas (R) occupy the largest portion of the region Public management and service facilities (A) are dispersed throughout the area, frequently located adjacent to commercial service facilities (B). Industrial areas (M) are primarily concentrated in the northwest, north, and northeast regions. We selected grids with high PDI values (P4 and P5) to emphasize areas with significant population aggregation. As presented in **Fig 6**, the degree of population aggregation was relatively low during the morning peak on February 18, with a scattered population distribution. However, population concentration on March 17 was significantly higher during the morning rush hour compared with February 18. Furthermore, the degree of population aggregation on March 16 exceeded both the evening peak of February 17 and March 16. In this region, functional areas with high PDI values are primarily industrial areas (M) and transportation and road facilities (S). The limited number of high PDI grids in the southeast of Xiamen Island can be attributed to the presence of natural scenic areas and Xiamen University. During the pandemic, these areas might have seen a decrease in tourism due to travel restrictions and safety concerns. Moreover, with students delaying their return to school, the student population in the region would have likely been lower than usual, contributing to the reduced population density.

Levels of population concentration, we performed a statistical analysis on the quantity of urban functional areas that fell into each category (**Fig A8 in S1 Appendix**). As depicted in **Fig A8 in S1 Appendix**, most of the population on February 17 was concentrated in the zones labeled among MP1, SP1, SP2, SP3, SP4, and SP5, with the highest density in transportation and road facilities areas. During the evening peak on both February 17 and March 16, there were more zones with concentrated populations on March 16, particularly in commercial service facility land, industrial land, transportation and road facility areas, and residential land. For the morning peak on February 18 and March 17, the number and types of functional areas with population gatherings on March 17 were notably larger than those on February 18.

**Fig 7:**
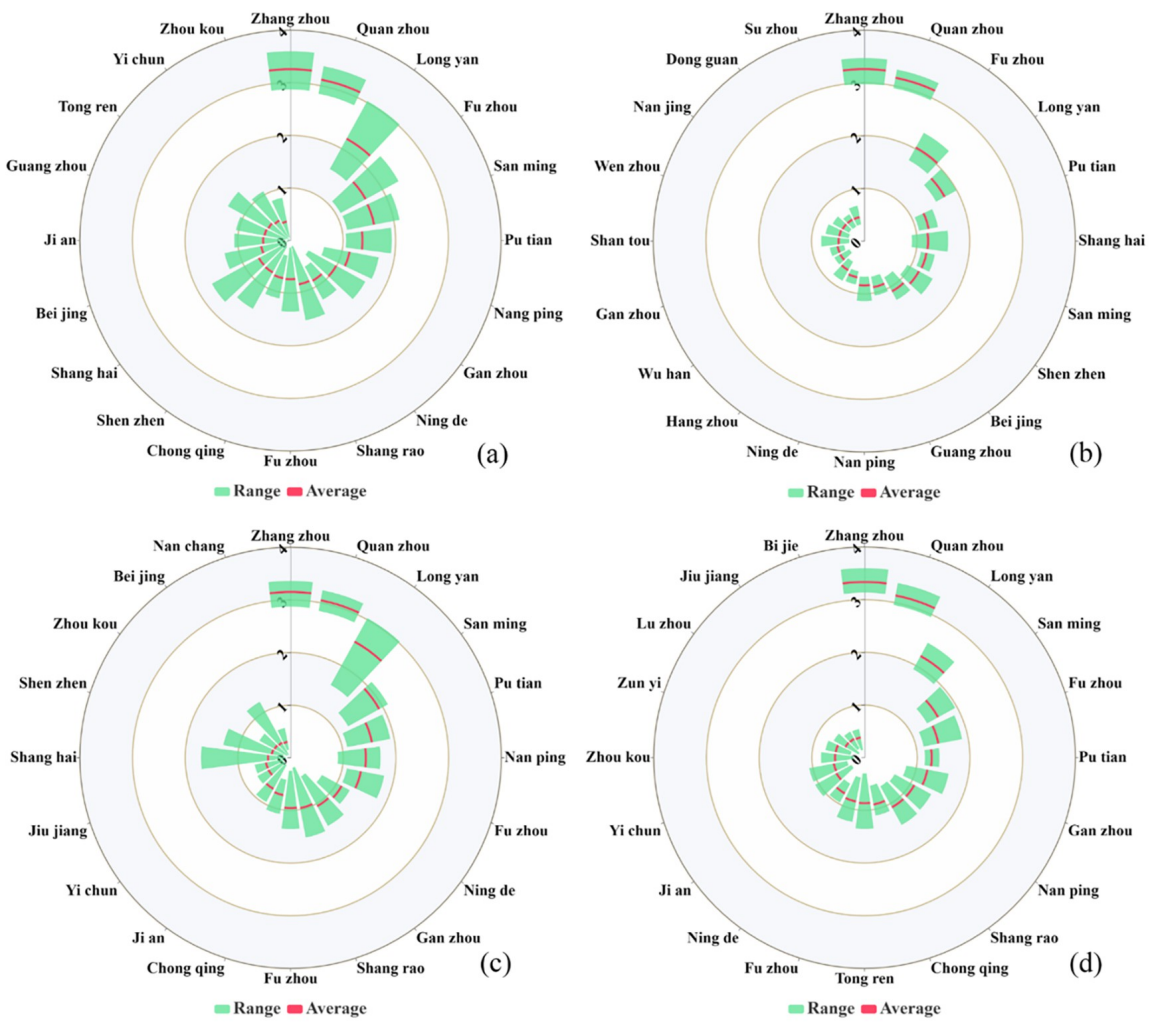
Nightingale rose diagram depicting Baidu migration and immigration indices during different periods in Xiamen (a: Jan 1–Mar 15, b: Jan 1–Jan 22, c: Jan 23–Feb 15, d: Feb 16–Mar 15). The green area signifies the range of the index, while the red line indicates the mean of the migration index.

Interurban migration significantly influences population dynamics, hence is an important factor in demographic changes. In the study, we conducted an analysis of population dynamics in the study area, with a primary focus on migration and emigration indices during the COVID-19 outbreak. As illustrated in **Fig 7**, the primary migration cities for Xiamen’s population are Zhangzhou, Quanzhou, and Longyan. This trend may be attributed to the geographic proximity of these cities to Xiamen and their economic and societal ties. Prior to and following the outbreak in 2020, the key migration cities for Xiamen were Zhangzhou, Quanzhou, Longyan, Fuzhou, Sanming, Putian, Nanping, and Ningde in the Fujian Province, all of which are adjacent to Xiamen. As demonstrated in **Fig 7C**, the migration index of all major cities decreased, with the average migration index of cities outside the province approaching zero. This decline indicates that the government’s lockdown policy significantly influenced migration trends. **Fig 7D** reveals that the migration indices of Xiamen, Quanzhou, and Zhangzhou increased relative to a certain period, while the migration indices for other cities, especially those within the province (Shang Rao, Chong Qing, Zhou Kou, and Yi Chun), trended toward zero.

**Fig 8:**
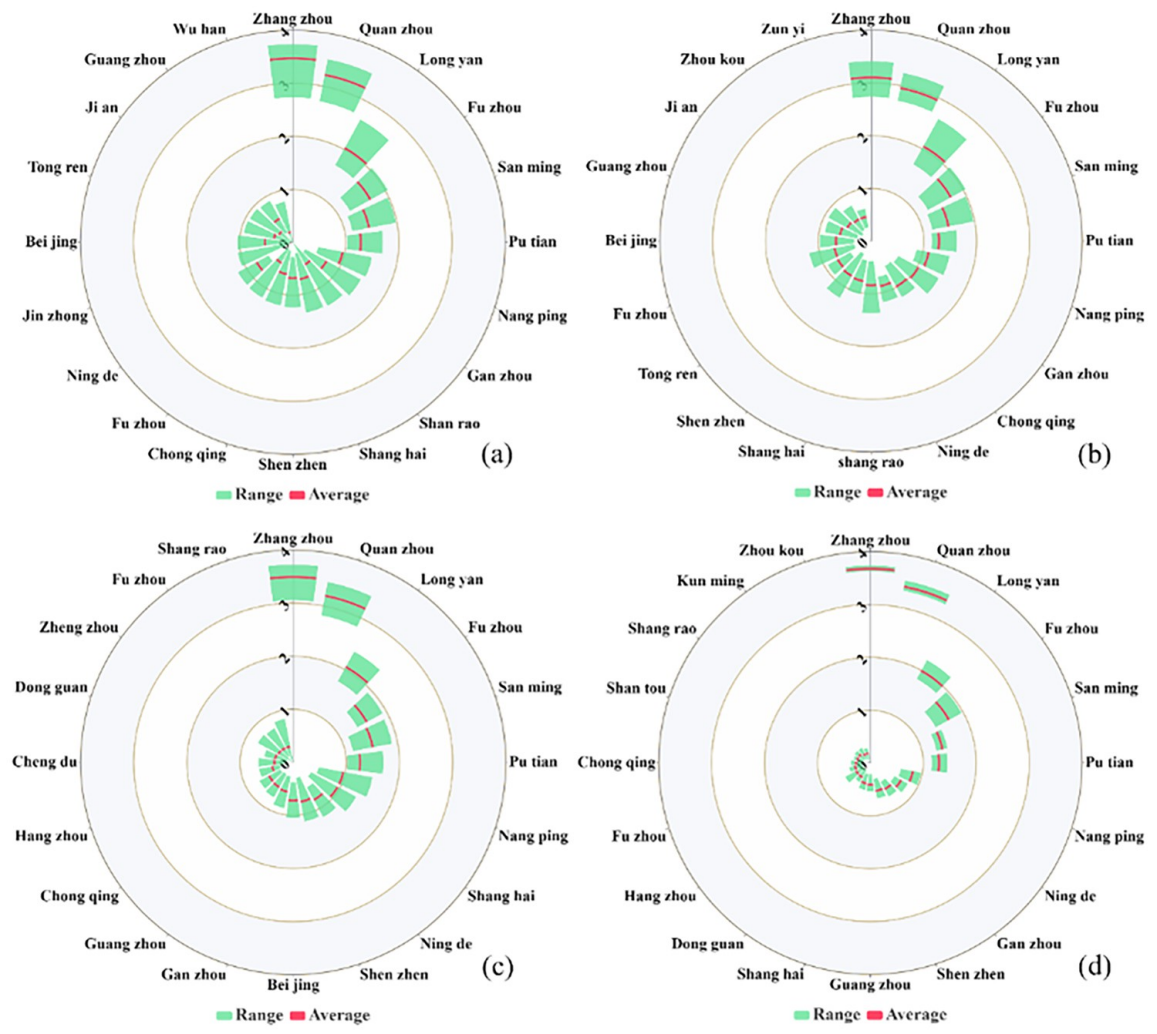
Nightingale rose diagram illustrating the variation in Baidu migration and emigration indices during different periods in Xiamen (a: Jan 1–Mar 15, b: Jan 1–Jan 22, c: Jan 23–Feb 15, d: Feb 16–Mar 15). The green area indicates the range of the index, while the red line signifies the mean migration index for each period.

**Fig 8** reveals that Zhangzhou, Quanzhou, and Longyan are the primary cities from which Xiamen’s population emigrates. **Fig 8B** portrays the period before the implementation of the policy designed to reduce population outflow. The mean population emigration index from Xiamen to Zhangzhou is intermediate, while that for Xiamen to Quanzhou and Longyan is closer to the minimum, and the indices to other cities are intermediate. **Fig 8C** represents the period from the commencement of the population outflow reduction policy to the onset of work resumption. During this phase, the population emigration indices from Xiamen to major cities in Jiangxi Province decreased. **Fig 8D** shows that Zhangzhou and Quanzhou remained the primary cities for population exodus from Xiamen, and the average, maximum, and minimum values of the emigration indices of these two cities are similar. The proportion of individuals relocating from Xiamen to other parts of the province was close to zero.

## 4 Discussion

### 4.1 Effectiveness of the proposed method

In this study, we analyzed the population distribution and investigated the influential factors on population dynamics during the pandemic. The robustness of our framework is substantiated through its application in analyzing population dynamics at various epidemic stages on Xiamen Island. Our developed framework utilizes hourly Baidu population heatmap data, employs programming technologies for spatial corrections, generates population heatmap data, and ascertains population aggregations at different spatial locations. This approach effectively identifies areas of population concentration. This study addresses the following key questions often posed by decision-makers: How is population distribution spatially and temporally influenced during a coronavirus outbreak? What are the projected infection numbers? What are the migration patterns during a pandemic? How has the spread of COVID-19 changed before and after work resumed?

Our proposed methodology offers several advantages: Firstly, it uses multi-source, extensive data to monitor and evaluate aspects related to spatiotemporal population dynamics. Secondly, it facilitates the study of population distribution during natural and epidemic disasters, including COVID-19. The results of our population analysis provide critical data to better facilitate government disaster response and mitigation strategies. Finally, the integration of spatiotemporal population distribution, migration, and POI-based urban functional zoning offers a comprehensive approach to studying the relationship between humans and their environment. This information provides essential guidance for decision-makers to better formulate their COVID-19 response strategies. Moreover, the framework’s simplicity makes it easily interpretable.

Xiamen Island, with a population density of 12,931 people/km^2^, was selected for the study. Its role as a major port, city center, and popular tourist destination on China’s southeast coast contributes to its highly dynamic population. As an internationally influential gateway city, Xiamen ranks 94^th^ globally and 9^th^ domestically for urban sustainable competitiveness. Considering these characteristics, the findings of our study apply to other cities and countries that share similar urban development features. Cities like Beijing, Shanghai, Hangzhou, Guangzhou, Singapore, and Tokyo, characterized by high population density and mobility, could potentially benefit from the findings reported herein. The relevance of this study extends to all urban areas facing similar challenges in managing population dynamics, urban planning, and response strategies during crisis periods or times of high mobility.

### 4.2 Patterns of population distribution

Early research has shown that the spread of COVID-19 is influenced by a population’s spatial distribution [77, 78]. The novel coronavirus typically follows a spatiotemporal evolution pattern, based on human interactions and activities [79]. This study delved into the population dynamics of Xiamen Island during various stages of the COVID-19 pandemic, focusing on both grid and community scales.

As depicted in **Fig A4 in S1 Appendix**, the spatiotemporal distribution of the population has undergone significant shifts over time. We determined that the population was predominantly concentrated in the central, southwest, east, and north areas of Xiamen Island, with fewer individuals spread throughout the coastal and southern regions. This distribution primarily results from government policies that promote a delayed return to work and enforce stay-at-home measures to reduce population mobility and concentration, thus effectively minimizing the risk of infection [17, 42, 80]. **Fig 2** reveals that the COVID-19 epidemic in Xiamen during 2020 was mainly concentrated in the Siming District. According to the heatmap and administrative zoning map of Xiamen Island, the population density was higher in the northern part of the Siming District and the southern part of the Huli District. Specifically, the Zhonghua and Xiagang communities exhibited notably high population concentration. Moreover, population density one month after work was resumed exceeded that of the first week post-resumption, with a larger area exhibiting high population density (**Fig A5 in S1 Appendix**). The results highlight that population dynamics undergo significant changes at both the grid and community scales during an epidemic period.

### 4.3 Main impact factors of population dynamics

Several studies have underscored the fundamental importance of managing the temporal and spatial dynamics between humans and the virus in the context of epidemic prevention and control [46, 47, 81, 82]. We investigated the impact of epidemic prevention policies, urban functional areas, and inter-city relations on population dynamics. As depicted in **Figs 3–5**, the enforcement of varying quarantine measures inevitably impacts the number of COVID-19 infections. Simulations using the SEIR model demonstrated that early implementation of home quarantine measures effectively reduced population aggregation, thus lowering infection risk during the initial stages of the coronavirus outbreak (**Figs 3–5**). While isolation policies contribute to epidemic control during high-infection periods, they invariably affect the economy [66].

Different urban functional areas exhibit diverse population distributions, and hot and cold spots emerge at various periods [42]. For instance, compared with industrial areas, commercial areas tend to have denser populations [42]. Data on population dynamics at the scale of urban functional areas can guide epidemic prevention in different governmental departments. **Fig 6** shows population-concentrated efforts across residential and transportation areas due to home quarantine measures and the delayed resumption of work. Approximately 1.5 months after work was resumed, the population distribution underwent significant shifts, with higher concentrations observed in residential, industrial, and transportation and road facility areas. Further, the population density one month post-resumption of work exceeded that within the first week, with a broader area experiencing higher population density.

The movement of individuals is inevitable as they live and work in cities and economic development progresses [16, 33]. This inter-city population movement drives dynamic population changes [83, 84]. However, these spatiotemporal changes vary, given the distinct spatial locations and economic and cultural relationships between cities. Our results highlight that population migration plays a crucial role in SARS-CoV-2 spread. As shown in **Figs 7 and 8**, Xiamen’s neighboring cities are the primary immigration and emigration sources. Following the COVID-19 outbreak, the migration indices of cities outside the province approached zero. Therefore, preventative efforts should target cities with high population migration indices to reduce inter-city transmission. Prior to the outbreak, most individuals traveling to and from Xiamen came from nearby cities. In contrast, after the outbreak, this proportion markedly decreased due to the transportation emergency policies enacted by relevant departments. These government measures successfully reduced gatherings and population movements, thereby minimizing transmission risks.

### 4.4 Management implications and limitations

To control the epidemic spread and mitigate its impact on the quality of life and economic development, we propose the following strategies to enhance epidemic prevention and control: First, a model was developed to explore the interaction between individual behavior, socio-economic factors, and the epidemic [85, 86]. This model highlights the importance of integrating social and economic development with the quality of life, as mentioned in other studies, in the context of epidemic prevention and control [87, 88]. Second, integrated monitoring and assessment of population dynamics and policy measures for epidemic prevention and control are crucial. This approach facilitates a comparative analysis of the epidemic’s effects on human health, socio-economic conditions, and urban development [89–91].

This endeavor should leverage technology such as the Internet, the Internet of Things, cloud computing, artificial intelligence, machine learning, and big data analytics [87, 92, 93]. Third, the development of more effective and practical prevention and control measures should be prioritized to mitigate the impact on socio-economic development, quality of life, and urban construction [94, 95]. Numerous studies have evaluated the epidemic control [88, 96, 97]. The integration of high-resolution spatial and temporal data on population movements will aid in formulating quarantine policies. Lastly, the provision of training and guidance to residents on epidemic prevention and control in their daily lives and work will help to reduce the spread. Effective prevention and control of the epidemic can be achieved through a better understanding and management of virus-policy interactions [47].

This study has some limitations. The Baidu population thermal map data does not represent the actual population values, instead shows the relative values of spatial population distribution. Similarly, Baidu migration data provide an index of individuals moving in and out of a particular city over a specific period, rather than the actual number. Some studies have utilized mobile signal data to overcome this limitation [98, 99]. Another limitation pertains to the SEIR model used in this study. Despite some discrepancies in its predictive capability for the number of infected individuals, the SEIR model can accurately characterize the impact of policy on infection trends [73]. Despite these limitations, this method holds significant potential as an effective and practical tool for providing real-time data to strategize the prevention and control of SARS-CoV-2 outbreaks.

## 5 Conclusion

Understanding the dynamic evolution of population characteristics during the novel coronavirus epidemic and the impact of various factors on viral transmission is essential for implementing effective and targeted prevention and control measures at different stages of the outbreak. Prior studies investigating population dynamics during the epidemic have encountered obstacles in acquiring accurate and timely population data. Additionally, several studies were limited in scope, typically examining the impact of population dynamics on virus infection from a single angle. To address these limitations, we employed a combination of multi-source data and model simulation methods to analyze the spatiotemporal dynamics of population distribution, as well as the primary factors influencing population dynamics during the epidemic. Our study findings suggest a significant correlation between the spread of SARS-CoV-2 and population dynamics. Our analysis demonstrates that various factors, including epidemic prevention and control policies, intercity population flows, and the distribution of different functional urban areas within cities, significantly impact population dynamics during the outbreak. In the absence of effective treatments for the novel coronavirus, population migration, and aggregation amplify the risk of viral transmission and infection due to the high transmissibility and infectious nature of the virus. Moreover, measures such as postponing the resumption of work and limiting gatherings in specific locations could help to reduce infection risks during the COVID-19 pandemic, despite their considerable impact on daily activities and economic development. To alleviate the epidemic’s impact on human life, the economy, and societal development, it is crucial to consider several factors, which include the severity of the epidemic, the dynamic characteristics of the population at various stages of development, and the state of economic and social development.

## Supporting information

S1 Text Data on Baidu migration and emigration across different time periods in Xiamen. (XLS)

S2 Text Data on Baidu migration and immigration across various time periods in Xiamen. (XLS)

S3 Text Statistics on novel coronavirus infections in cities across China from January 16 to March 15, 2020. (XLS)

S1 Appendix Contains the following Figs: Figs A1-A8. (DOCX)

S2 Appendix Contains the Table B1. (DOCX)
