# Supplementary material for: A data-driven framework to assess population dynamics during novel coronavirus outbreaks: A case study on Xiamen Island, China
Source: PLoS One. 2023 Nov 10;18(11):e0293803. doi: 10.1371/journal.pone.0293803 (PMC10637684; doi:10.1371/journal.pone.0293803)
Supplement: S1 Appendix — (DOCX) [file pone.0293803.s004.docx]

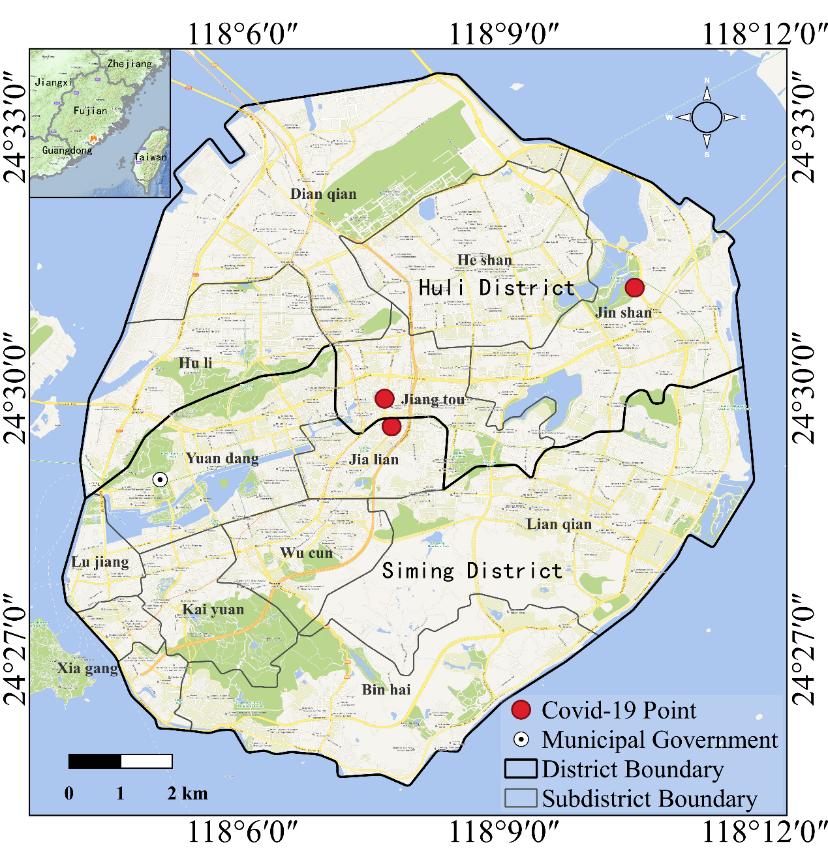


Fig. A1 Geographic representation of Xiamen Island


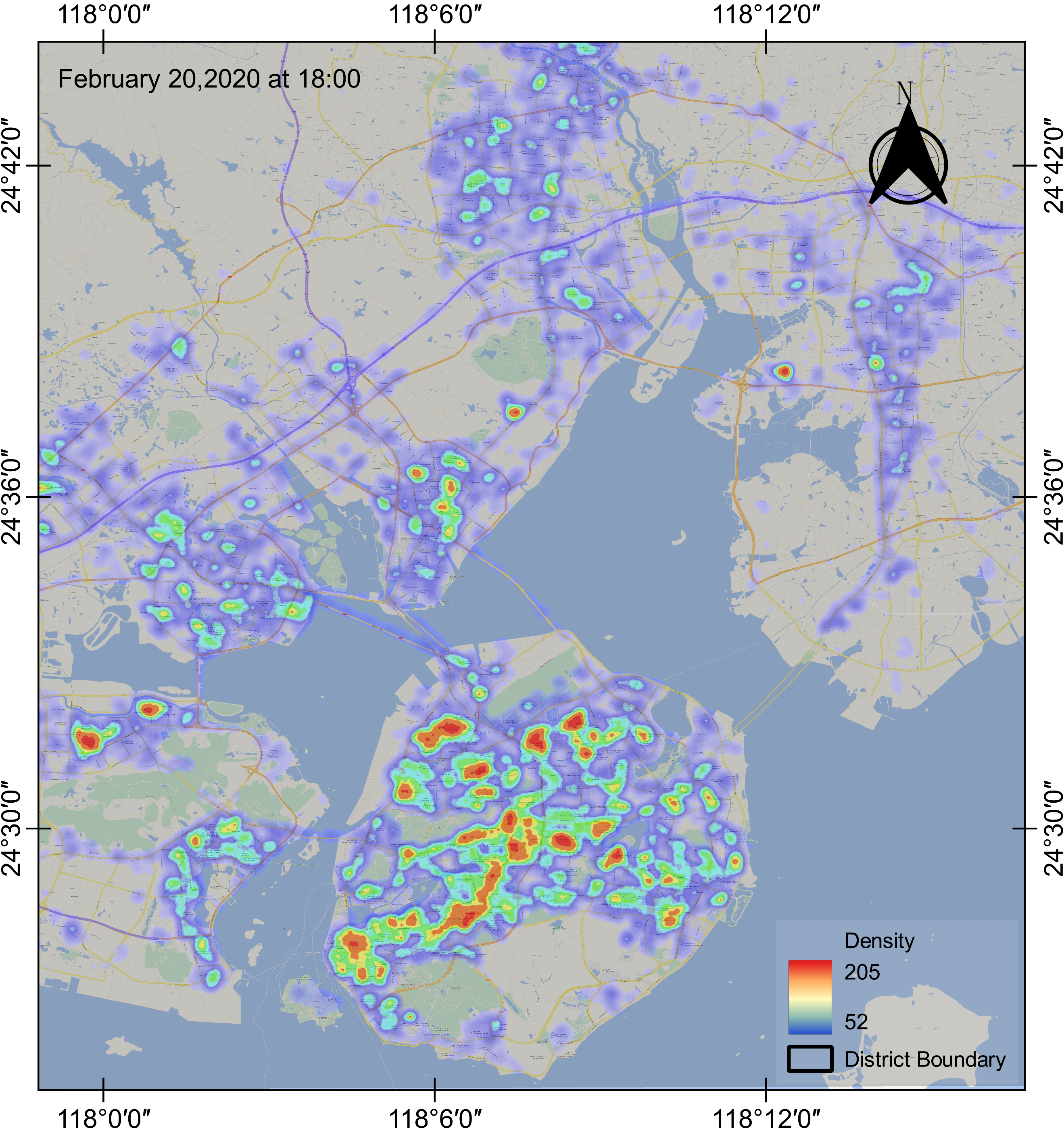


**Fig. A2** Spatial distribution of the population on Xiamen Island and its surrounding adjacent regions

**
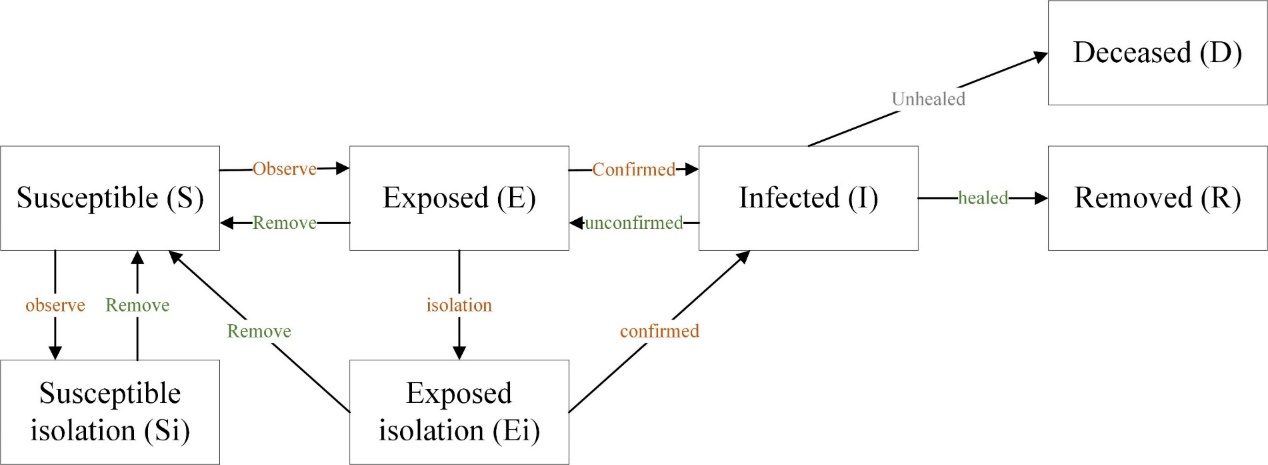
**

**Fig. A3** Conceptual diagram of the SEIR infection mechanism


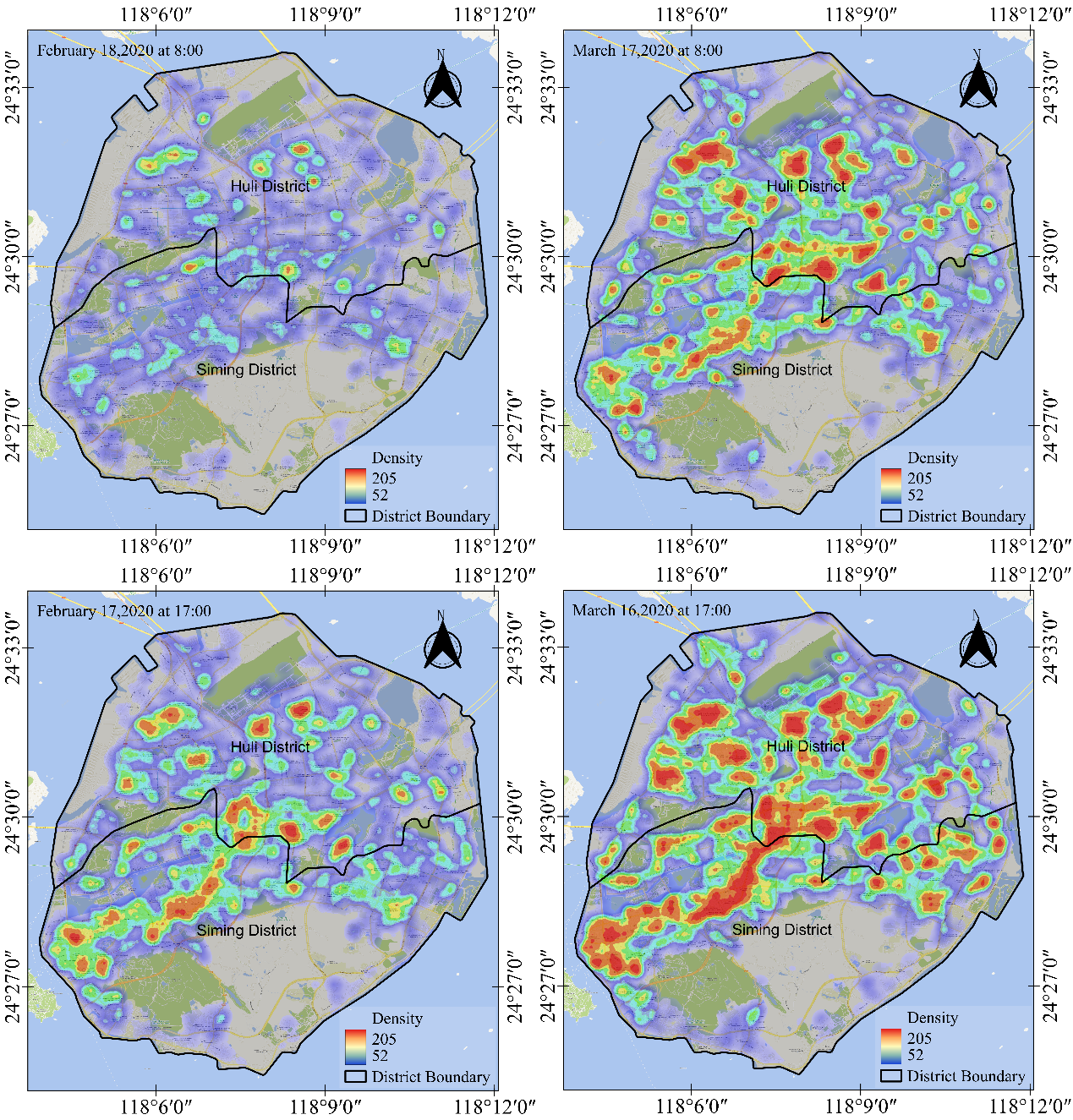


**Fig. A4** Baidu heat maps depicting Xiamen Island at various time periods


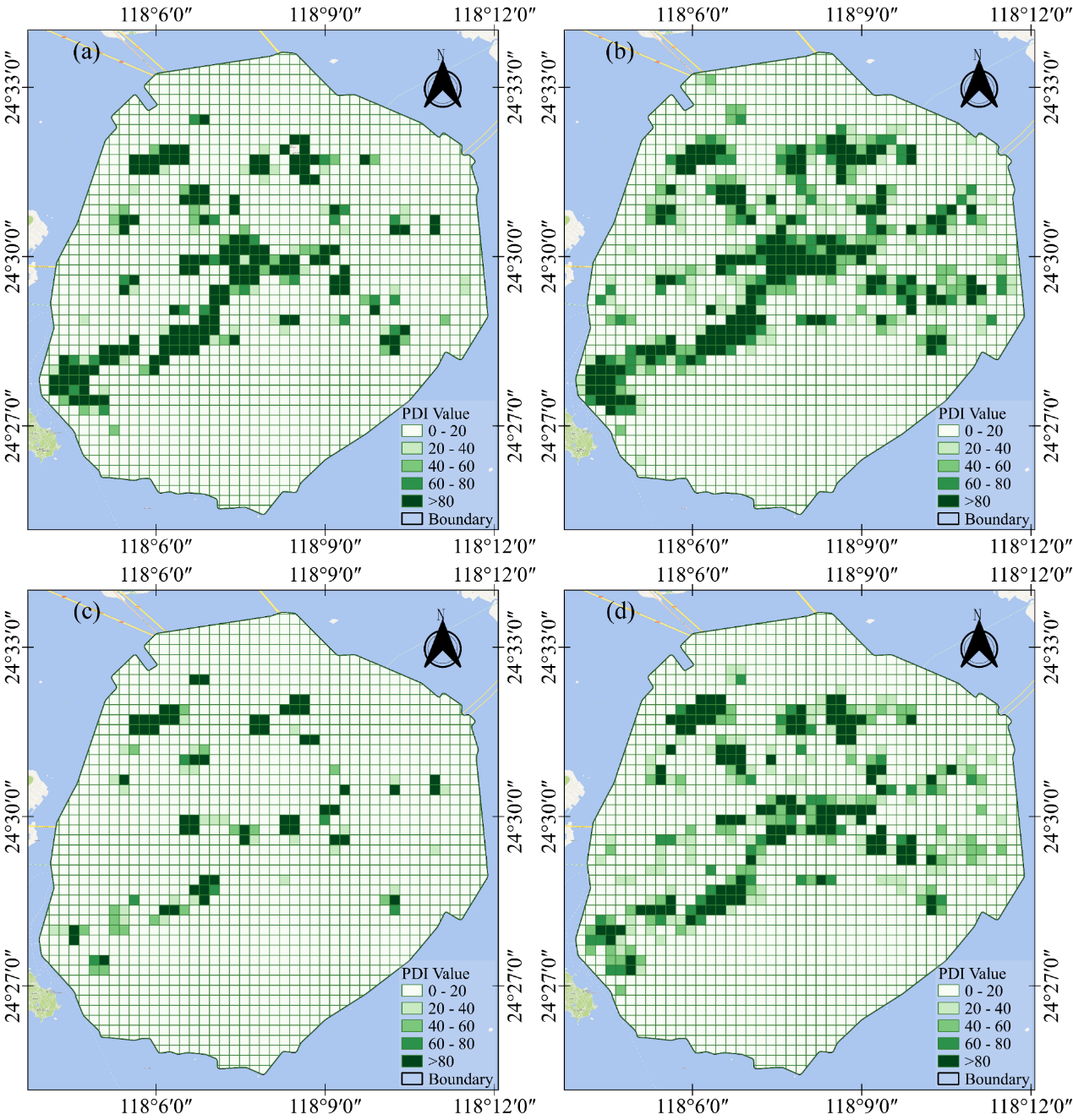


**Fig. A5** Spatial distribution of the population on Xiamen Island during different timeframes: (a) 17:00–19:59 on February 17, 2020, (b) 17:00–19:59 on March 16, 2020, (c) 7:00–8:59 on February 18, 2020, and (d) 7:00–8:59 on March 17, 2020.


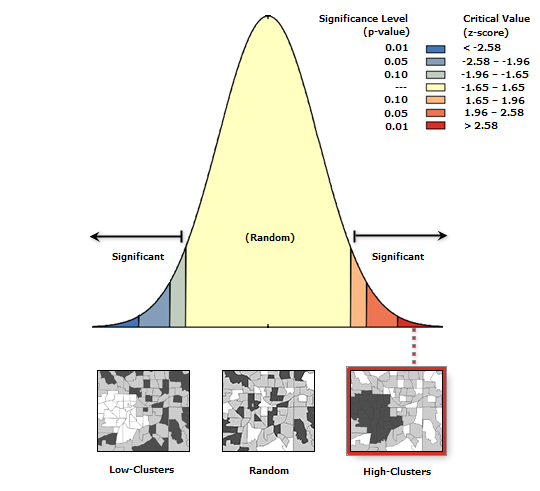


**Fig. A6** Illustration of the null hypothesis pertaining to the high/low clustering grid detection of the population [[69]](#_CTVL001a3a8c0d850be4509ac377271131def8a" \o "Arcgis Pro. 2019 (2019). Retrieved from https://​www.esri.com​/​zh-cn/​arcgis/​products/​arcgis-pro/​overview.)


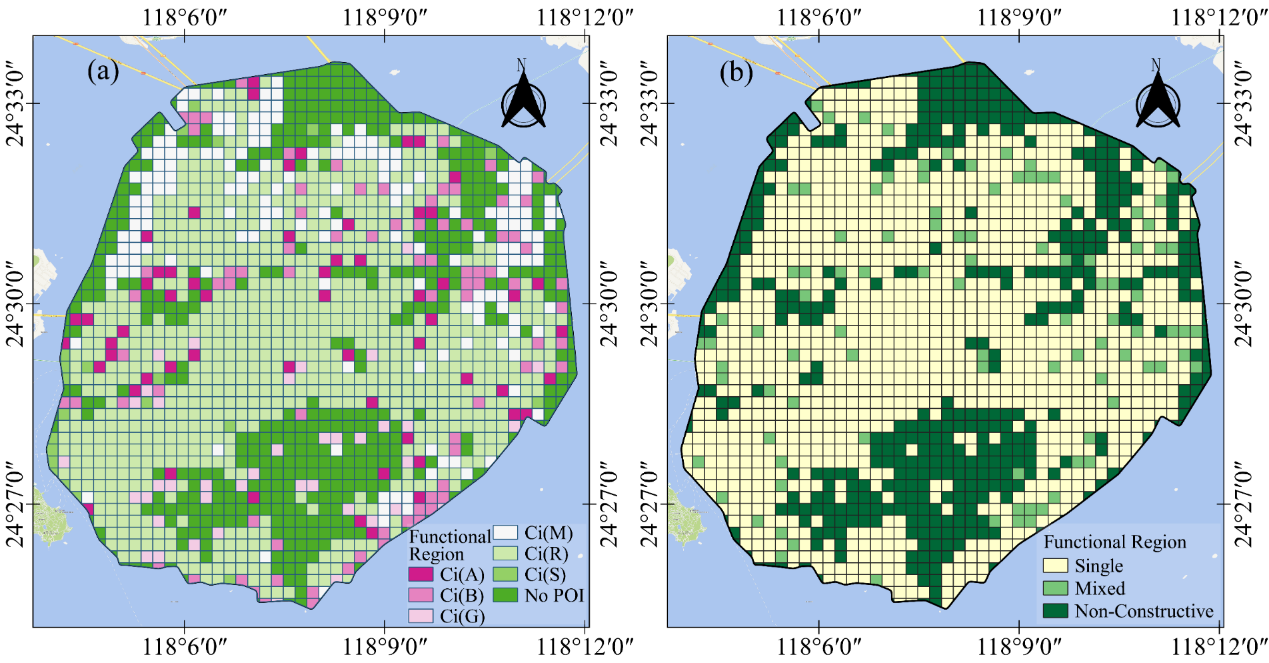


**Fig. A7** Spatial distribution of (a) urban and (b) different city functional zone types

**
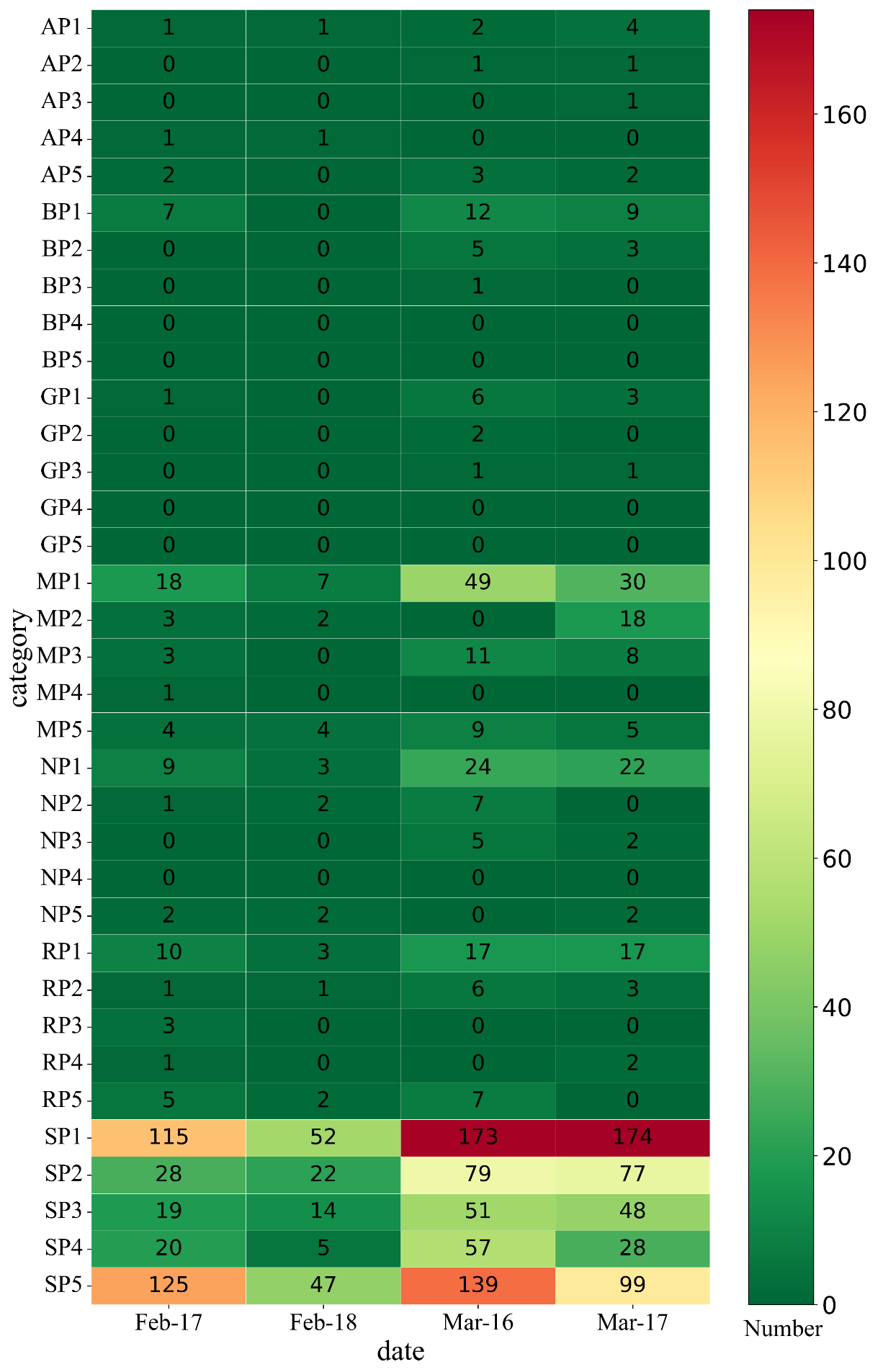
**

**Fig. A8** The count of urban functional zones under varying degrees of population aggregation
