# Supplementary material for: A data-driven framework to assess population dynamics during novel coronavirus outbreaks: A case study on Xiamen Island, China
Source: PLoS One. 2023 Nov 10;18(11):e0293803. doi: 10.1371/journal.pone.0293803 (PMC10637684; doi:10.1371/journal.pone.0293803)
Supplement: S2 Appendix — (DOCX) [file pone.0293803.s005.docx]

# Appendix B

**Table B1 Summary of High/Low Clustering Results Data**

| Time period | G (observed value) | G (expected value) | Z | P |
| --- | --- | --- | --- | --- |
| Feb–17 M | 0.00109 | 0.00075 | 13.15273 | 0.00 |
| Feb–17 E | 0.00100 | 0.00072 | 13.83146 | 0.00 |
| Feb–18 M | 0.00109 | 0.00075 | 13.06317 | 0.00 |
| Feb–18 E | 0.00100 | 0.00071 | 13.32953 | 0.00 |
| Feb–19 M | 0.00109 | 0.00076 | 12.81956 | 0.00 |
| Feb–19 E | 0.00100 | 0.00071 | 13.46056 | 0.00 |
| Feb–20 M | 0.00107 | 0.00075 | 13.36905 | 0.00 |
| Feb–20 E | 0.00098 | 0.00071 | 13.81832 | 0.00 |
| Feb–21 M | 0.00107 | 0.00074 | 13.33082 | 0.00 |
| Feb–21 E | 0.00098 | 0.00070 | 13.89206 | 0.00 |
| Mar–16 M | 0.00100 | 0.00073 | 15.28708 | 0.00 |
| Mar–16 E | 0.00097 | 0.00069 | 14.19889 | 0.00 |
| Mar–17 M | 0.00100 | 0.00074 | 15.13439 | 0.00 |
| Mar–17 M | 0.00097 | 0.00067 | 14.48898 | 0.00 |
| Mar–18 M | 0.00100 | 0.00074 | 15.03358 | 0.00 |
| Mar–18 M | 0.00099 | 0.00070 | 13.70464 | 0.00 |
| Mar–19 M | 0.00101 | 0.00073 | 15.30003 | 0.00 |
| Mar–19 M | 0.00098 | 0.00068 | 14.07602 | 0.00 |
| Mar–20 M | 0.00101 | 0.00073 | 15.36321 | 0.00 |
| Mar–20 M | 0.00095 | 0.00066 | 14.67839 | 0.00 |

Notes: M= Morning peak; E= Evening peak
